# Editorial: Pulsed Electric Fields in Biotechnology

**DOI:** 10.3389/fbioe.2021.639150

**Published:** 2021-04-30

**Authors:** Saša Haberl Meglič, Eugene Vorobiev, Damijan Miklavčič

**Affiliations:** ^1^Faculty of Electrical Engineering, University of Ljubljana, Ljubljana, Slovenia; ^2^University of Technology Compiegne, Compiègne, France

**Keywords:** extraction of value compounds, product modification and properties, modeling and numerical simulations, PEF bioprocessing, equipment design in biotechnology, pulsed field ablation

Almost five decades ago it was shown that the permeability of a vesicular membrane can be increased by exposure to electric pulses. Today we know that when we expose the cell membrane to electric pulses of adequate strength and induced transmembrane voltage surpasses a certain value, the cell membrane becomes permeable. Thus, small or large molecules can be introduced into or extracted from cells. The phenomenon is called electroporation and is today showing remarkable promise in various fields: medicine, biology, and food- and biotechnology.

Especially applications of electric pulses in biotechnology have attracted much attention over recent years. Pulsed electric fields (PEF) in biotechnology are being rapidly developed and used in a wide range of areas: exploitation of microorganisms for producing value compounds, manufacture of biofuels from renewable feedstocks, removal pathogenic microorganisms from various water sources (i.e., hospital wastewaters), and liquid foods where vitamins and the food's color, flavor, or texture is unaffected. Nevertheless, a vast amount of work still needs to be done.

As the population grows, so does the need for a variety of dietary supplements and high-quality value compounds. Therefore, researchers began to look for new ways to obtain these compounds. One possible way is to grow microorganism cells that can produce these for us. Several physical or chemical methods are already established in order to disrupt microorganism cells and collect their content. Nevertheless, the main drawbacks of many chemical methods are the use of expensive chemicals and the necessity of removing them from the final product. Furthermore, on the pharmaceutical production scale, the use of such chemicals is restricted by regulatory bodies. The largest drawback of physical methods is the extensive fragmentation of the microorganism, which requires a costly downstream purification process. Consequently, the cost of value compounds' extraction remains high, providing strong motivation for new extraction tools and procedures. A Research Topic on Pulsed Electric Fields in Biotechnology is therefore aimed at providing a comprehensive update on the extraction of value compounds by means of electroporation from microorganisms. Since electroporation is affecting every cell membrane, assortments of microorganisms are being presented where proteins, carbohydrates, and dyes are being extracted by means of electroporation. The most important advantage when using electroporation compared to other established methods for extraction was found to be the lack of co-extracting impurities (Haberl Meglič et al.; Eleršek et al.; Ganeva et al.). Although extraction by means of electroporation largely avoids total cell disintegration with which the extract is a mix of extracted compounds with debris, the protocol still needs to be optimized in order to obtain a higher yield. Thus, a combination of electroporation with other methods, such as mild heating, could represent a suitable approach for the efficient recovery of value compounds (Carullo et al.). Furthermore, the optimization of solvent media is crucial in order to obtain the wanted compound from the microorganism, and the extraction of the antioxidant can be mediated by enzymatic esterase activity triggered by PEF (Aguilar-Machado et al.). Additionally, more accurate microdosimetric numerical models of cells are needed in order to investigate the electroporation phenomenon and to more precisely set up efficient and controlled electroporation protocol. That can be achieved by building a realistic model of biological cells using optical microscopy images with highlighted cell compartments (De Angelis et al.).

In order to facilitate PEF application on a large scale, the development of continuous treatments has been pursued. A standard PEF continuous treatment system therefore consists of a pulse generator that enables continuous pulse treatment, flow chambers with electrodes, and a fluid-handling system. In order to overcome hurdles in PEF continuous systems such as inhomogeneous treatment conditions, a treatment chamber with more homogeneous flow properties inside is presented (Schottroff et al.). Since there is also a difficulty in comparing data obtained in different chambers or at different scales, kinetic modeling, and numerical simulations of treatment chambers are needed and also presented within this topic (Knappert et al.).

In the food industry, PEF shows great promise especially in the extraction of juice from fruits and vegetables. Namely, standard methods (enzymatic, mechanical, or thermal treatment) can cause a loss in juice quality (loss of vitamins *etc*.) and taste due to heating or enzymatic activity.

Hops are the most complex and costly raw material used in the brewing industry since they give the beer its flavor and also preserve the beverage. Although different brewing technologies are used in order to enhance the extraction of acids from hops, the possibility of using PEF for this purpose was also presented for the first time (Ntourtoglou et al.).

Although the topic mainly focuses on electroporation in biotechnology, also remarkable achievements can be found in medicine. There is an increasing interest in using biocompatible nanotechnologies in medicine, combined with the electric field, which can be used as drug delivery systems. Thus, the likelihood of controlling the release of drugs from liposome vesicles using electric fields is presented. The study's experimental results are supported by simulations in order to reveal the accompanying interactions and to optimize the electroporation protocol (Caramazza et al.). Also, the characterization of conductivity changes induced by electroporation using all treatment pulses is presented in order to simulate the electroporation process more precisely. Such a model is vital for treatment planning in medicine where electroporation is used (Zhao et al.).

A very new technology that will with great certainty completely change cardiac ablation is pulsed-field atrial and ventricular myocardial ablation. Presented herein is a proof-of-concept work with clearly stated claims of feasibility and safety. Nevertheless, further and systematic preclinical quantification both in the atrial and ventricular myocardium is necessary before stepping into the clinical validation of non-inferiority (Caluori et al.).

This Research Topic collected selected contributions from participants of the third World Congress on Electroporation, Pulsed Electric Fields in Biology, Medicine, Food and Environmental Technologies, held in Toulouse, France, on September 3–6, 2019 (https://wc2019.electroporation.net/).

## Author Contributions

SHM drafted the manuscript. DM and EV critically revised the manuscript. All authors read and approved the submitted manuscript.

## Conflict of Interest

The authors declare that the research was conducted in the absence of any commercial or financial relationships that could be construed as a potential conflict of interest.

